# Cost-Effectiveness of Team-Based Coaching With Surveillance for Prevention of Acute Kidney Injuries

**DOI:** 10.1001/jamanetworkopen.2025.2503

**Published:** 2025-04-02

**Authors:** David Xiao, Sharon E. Davis, Caroline M. Godfrey, Hanxuan Yu, Elizabeth Sullivan, Jinyi Zhu, Ashley A. Leech, Kevin C. Cox, Iben Ricket, Michael E. Matheny, Jeremiah R. Brown, Stephen A. Deppen

**Affiliations:** 1 Department of General Surgery, Vanderbilt University Medical Center, Nashville, Tennessee; 2 Department of Department of Biomedical Informatics, Vanderbilt University Medical Center, Nashville, Tennessee; 3 Department of Health Policy, Vanderbilt University Medical Center, Nashville, Tennessee; 4 Department of Epidemiology, Geisel School of Medicine at Dartmouth, Hanover, New Hampshire; 5 Department of Thoracic Surgery, Vanderbilt University Medical Center, Nashville, Tennessee; 6 Department of Biostatistics, Vanderbilt University Medical Center, Nashville, Tennessee; 7 Department of Medicine, Vanderbilt University Medical Center, Nashville, Tennessee; 8 Geriatrics Research Education and Clinical Care, Tennessee Valley Healthcare System VA, Nashville; 9 Dartmouth Center for Implementation Science, Departments of Epidemiology and Biomedical Data Science, Geisel School of Medicine at Dartmouth, Hanover, New Hampshire

## Abstract

**Importance:**

More than 10% of US patients undergoing endovascular procedures experience contrast-associated acute kidney injuries (AKIs), resulting in increased costs and health deficits. Prevention protocols reduce AKIs, but uptake and adherence vary greatly, and the cost-effectiveness of these interventions is unknown.

**Objective:**

To analyze the cost-effectiveness of 4 implementation interventions for AKI prevention in patients undergoing cardiac catheterizations.

**Design, Setting, and Participants:**

This economic evaluation used a Markov decision model with 3-year horizon was constructed to simulate quality-adjusted life years (QALYs) and costs after AKI prevention protocol implementation for patients undergoing cardiac catheterization. Data from the IMPROVE AKI trial, a cluster-randomized trial conducted across 20 US Department of Veterans Affairs medical centers from 2019 to 2021, were used for probabilities, with economic and utility data derived from literature. Patients aged 18 years or older, who underwent cardiac coronary angiography for diagnostic or treatment of pathology were included. Patients with a history of dialysis (hemodialysis or peritoneal dialysis) were excluded. Data were analyzed from January to June 2024.

**Exposure:**

Interventions compared were assistance, assistance with surveillance, collaborative, and collaborative with surveillance.

**Main Outcomes and Measures:**

QALYs and cost in dollars discounted at 3% per year and incremental cost-effectiveness ratio (ICER) using willingness-to-pay threshold of $100 000 per QALY. One-way and probabilistic sensitivity analyses were performed.

**Results:**

Among 122 803 patients, 13 047 experienced AKIs (10.6%). Patient characteristics were balanced across 4 groups with an overall median (IQR) age of 70 (65-74) years, 119 119 males (97%), 25 789 Black patients (21%), 88 418 White patients (72%), and 8596 for all other racial and ethnic groups (7%). AKI incidences were 13.3% (95% CI, 11.0%-15.6%) in assistance, 11.4% (95% CI, 9.5%-13.3%) in assistance with surveillance, 12.7% (95% CI, 11.1%-14.4%) in collaborative, and 7.9% (95% CI, 6.4%-9.5%) in collaborative with surveillance. Intervention costs per patient were $12.74 (IQR, $9.56-$15.93) for collaborative with surveillance, $3.97 (IQR, $2.98-$4.96) for collaborative, $3.36 for assistance with surveillance, and $2.69 for assistance. Drivers for total cost of interventions were costs of AKI and subsequent permanent kidney disease. ICERs revealed collaborative with surveillance as economically dominant. Compared with assistance, collaborative with surveillance saved $742.75 while improving cost-effectiveness by 0.02 QALYs per person. Results were robust to sensitivity analyses.

**Conclusions and Relevance:**

In this economic evaluation of implementation strategies for AKI prevention, virtual learning collaborative with automated surveillance reporting was the economically preferred intervention and was estimated to decrease AKI likelihood, permanent kidney disease, and their associated costs after undergoing cardiac catheterization. These results may be generalizable to other endovascular procedures and practice-changing protocols or checklists implementation efforts.

## Key Points

**Question:**

What is the cost-effectiveness of interventions for implementing protocols for preventing contrast-associated acute kidney injuries (AKI) in patients undergoing cardiac catheterization?

**Findings:**

This economic evaluation of 122 803 patients used a Markov decision model and found that virtual learning collaborative with automated surveillance reporting was the most cost-effective implementation strategy for AKI prevention, resulting in lower estimated AKI incidences and permanent kidney disease, which led to reduced associated costs.

**Meaning:**

These findings suggest that implementing virtual learning collaborative with automated surveillance reporting for AKI prevention protocol implementation is the most economically favorable intervention and may be applicable to other endovascular procedures and could influence broader practice changes.

## Teaser

This economic evaluation used a Markov decision model to analyze the cost-effectiveness of 4 implementation interventions for acute kidney injury prevention in patients undergoing cardiac catheterizations.

## Introduction

Endovascular procedure volumes have increased steadily across many medical disciplines, especially as endovascular techniques continue to advance. The minimally invasive percutaneous approach can be used for cardiac catheterizations, cardiac valve replacements, aortic aneurysm repairs, revascularization of peripheral vascular disease, neurovascular diseases, chemoembolization of solid tumors, and more. Diagnostic and interventional cardiac catheterizations alone account for more than 1 million procedures a year performed in the US.^1^ One of the most common complications arising from endovascular procedures is contrast-associated acute kidney injuries (AKIs), which are associated with an increased risk of permanent loss of kidney function, cardiovascular events, mortality, and prolonged hospitalization. All generate additional short- and long-term costs.^2,3,4^

Increasing intravenous volume and limiting contrast volume are 2 widely accepted and evidence–supported broad approaches to prevent contrast–related AKI.^5,6,7^ Many detailed checklists for these guideline-based protocols are available for clinical implementation, but their uptake and adherence vary significantly across care teams and institutions, leading to suboptimal care in many settings.^6^ One promising institutional implementation strategy, developed at the Veterans Affairs Medical Center (VAMC), is the virtual learning collaborative, which involves collective problem-solving using quality improvement methods, such as Six Sigma, Lean, PDSA cycles, in the process of implementing complex practice-changing protocols.^8^ The virtual aspect of virtual learning collaborative refers to resources such as voice or video teleconferencing, which maximizes access and scalability by reducing the costs and logistical challenges associated with in-person learning collaboratives. In contrast, traditional practice-changing protocol implementation strategies involve having site-specific training and time-limited technical assistance done virtually or in person, but without training and use of quality improvement methods.

A regional pilot multisite implementation study demonstrated that the use of virtual learning collaborative strategies led to a 21% reduction in AKIs overall and a 28% reduction among patients with preexisting chronic kidney disease (CKD) compared with standard of care.^9^ This study’s success led to the IMPROVE AKI trial.^7^ Briefly, IMPROVE AKI was a large multisite, 2 × 2 factorial cluster-randomized trial evaluating 4 different implementation strategies in the prevention of AKIs among patients undergoing cardiac catheterizations at VAMCs. Twenty VAMC cardiac catheterization centers were randomized to 1 of 2 implementation strategies and to information support tools. All sites received an AKI prevention protocol with guidance on established practices for reducing AKI. Implementation strategies for deploying this protocol were technical assistance (ie, assistance) and team-based coaching through the virtual learning collaborative approach (ie, collaborative). For those sites randomized to receive information support tools (ie, surveillance), a dashboard was made available that provided temporal surveillance of AKI outcomes with site-specific risk-adjusted AKI performance over time and benchmarking insight against other VAMC centers. In addition to the primary outcome of AKI within 7 and 30 days, costs associated with training, reporting, checklist implementation, and other intervention–specific activities or personnel were collected.

 Ultimately, this study showed that the collaborative with surveillance approach was the most effective implementation strategy for preventing AKIs in patients undergoing cardiac catheterization. However, it also was associated with the most implementation costs and resources. We sought to compare the 4 intervention strategies to determine the most cost effective and drivers of costs in AKI prevention interventions.

## Methods

This economic evaluation did not require approval from the Vanderbilt University institutional review board or informed consent because the study used secondary data sources. We followed the Consolidated Health Economic Evaluation Reporting Standards (CHEERS) reporting guideline in reporting our economic analysis study (eTable in Supplement 1).

### Model Construction

**Figure 1.**
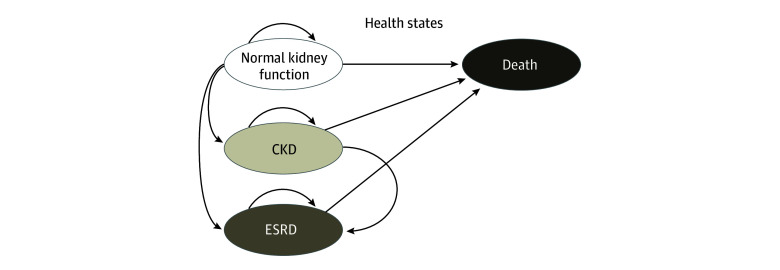
Schematic Representation of Markov State Diagram After Cardiac Catheterization Circles indicate various states and arrows indicate transitions between various states. CKD indicates chronic kidney disease; ESRD, end stage renal disease.

We constructed a cohort-based Markov model to assess the cost-effectiveness of the 4 IMPROVE AKI implementation strategies of AKI prevention: collaborative, collaborative with surveillance, assistance, and assistance with surveillance. Our model starts as a decision tree with 4 branches off of the initial decision node for each of the 4 interventions, with each implementation strategy having 2 chance nodes of developing AKI and not developing AKI at day 7, followed by probabilities of being in 1 of 4 health states: normal kidney function, CKD, end stage renal disease (ESRD), and death at day 30. The model then transitioned to a Markov model with yearly cycles for a horizon of 3 years with transition probabilities between the aforementioned health states obtained from the IMPROVE AKI study (Figure 1). The yearly transition probability estimate used for the analysis was the mean of the 3 yearly transition probabilities observed in the study. Normal kidney function can stay constant or transition to CKD, ESRD, or death. CKD can stay constant or transition to ESRD or death. ESRD can stay constant or transition to death. The full decision tree followed by the Markov model is available in eFigure 1 in Supplement 1. We used trial data to inform transition probabilities.

### Study Population

Data were derived from the multicenter IMPROVE AKI trial, which collected data about all patients who underwent cardiac cauterizations at participating VAMCs from October 1, 2019, to March 31, 2021.^7^ All VAMCs with a cardiac catheterization laboratory were invited to participate. Twenty centers enrolled and were randomized to 1 of the 4 interventions. Patient eligibility included all patients aged 18 years or older, who underwent cardiac coronary angiography for diagnostic or treatment of pathology. Patients with a history of dialysis (hemodialysis or peritoneal dialysis) were excluded. 

### Model Inputs

AKI rates, state-transition probabilities, and the costs of each intervention, which consisted of costs associated with training and creation of digital reports when used, were estimated using IMPROVE AKI study trial data (Table 1). The proportion of patients in each trial group developing AKI was 13.3% (95% CI, 11.0%-15.6%) in assistance, 11.4% (95% CI, 9.5%-13.3%) in assistance with surveillance, 12.7% (95% CI, 11.1%-14.4%) in collaborative, and 7.9% (95% CI, 6.4%-9.5%) in collaborative with surveillance.^7^

**Table 1.** Input Variables and Range Tested for Sensitivity Analysis

Parameters^a^	Base case (95% CI), %	Source
**Acute kidney injury probabilities**		
Collaborative strategy (pAKI_Collab)	12.7 (11.1-14.4)	Trial data^7^
Assistance strategy (pAKI_Assist)	13.3 (11.0-15.6)	Trial data^7^
Collaborative with Surveillance Strategy (pAKI_Collab_Surv)	7.9 (6.4-9.5)	Trial data^7^
Assistance with Surveillance Strategy (pAKI_Assist_Surv)	11.4 (9.5-13.3)	Trial data^7^
**Transition probabilities at 30 d**		
AKI→Normal Kidney (pRecover)	86.9 (86.3-87.4)	Trial data^7^
AKI→ CKD (pCKD)	3.6 (3.3-3.9)	Trial data^7^
AKI→ ESRD (pESRD)	0.4 (0.3-0.5)	Trial data^7^
AKI→ Death (pDeathAKI)	9.1 (8.7-9.7)	Trial data^7^
No AKI→ Normal Kidney (pHealth)	97.3 (97.2-97.4)	Trial data^7^
No AKI→ CKD (pnewCKD)	1.2 (1.1-1.3)	Trial data^7^
No AKI→ ESRD (pnewESRD)	0	Trial data^7^
No AKI→ Death (pDeathNoAKI)	1.4 (1.4-1.5)	Trial data^7^
**Transition probabilities (±25%)**		
Normal Kidney (After AKI) →CKD (pRecovery_CKD)	2.8 (2.1-3.5)	Trial data^7^
Normal Kidney (After AKI)→ ESRD (pRecovery_ESRD)	0.3 (0.2-0.4)	Trial data^7^
Normal Kidney (After AKI) → Death (pRecovery_Die)	12.3 (9.2-15.4)	Trial data^7^
Normal Kidney (No AKI)→ CKD (pHealth_CKD)	1 (0.8-1.3)	Trial data^7^
Normal Kidney (No AKI)→ ESRD (pHealth_ESRD)	0.05 (0.04-0.06)	Trial data^7^
Normal Kidney (No AKI)→ Death (pHealth_Die)	6.7 (5.0-8.4)	Trial data^7^
CKD (After AKI)→ ESRD (pCKD_ESRD)	4.7 (3.5-5.9)	Trial data^7^
CKD (After AKI)→Death (pCKD_Die)	29.7 (22.3-37.1)	Trial data^7^
CKD (No AKI)→ ESRD (pCKD_ESRD_NoAKI)	1.8 (1.4-2.3)	Trial data^7^
CKD (No AKI)→ Die (pCKD_Die_NoAKI)	27.3 (20.5-34.1)	Trial data^7^
ESRD (After AKI)→ Death (pESRD_Die)	34.5 (25.9-43.1)	Trial data^7^
ESRD (No AKI)→ Death (pESRD_Death_NoAKI)	27.5 (20.6-34.4)	Trial data^7^
**Utility weights**		
Healthy (U_Healthy)	0.85 (0.83-0.87)	Mazta et al^10^
CKD (U_CKD)	0.80 (0.7-1.0)	Cooper et al^11^
ESRD (U_ESRD)	0.7 (0.6-0.8)	Cooper et al^11^
Death	0	
**Upfront costs per person (±25%), $**		
Collaborative Strategy (C_Collab)	3.97 (2.98-4.96)	Trial data^7^
Assistance Strategy (C_Assist)	2.69 (2.02-3.36)	Trial data^7^
Collaborative and Surveillance Strategy (C_CollabSurv)	12.74 (9.56-15.93)	Trial data^7^
Assistance and Surveillance Strategy (C_AssistSurv)	3.36 (2.52-4.20)	Trial data^7^
**Health state costs per person, (±25%), $**		
AKI cost, per episode (2020) (C_AKI)	14 000 (8200-13 750)	Subramanian et al^12^
CKD cost, yearly (2020) (C_CKD)	25 322 (18 992-31 653)	Medicare data^13^
ESRD cost, yearly (2020) (C_ESRD)	78 537 (58 903-98 171)	Medicare data^13^
Death	0 (0)	

Abbreviations: AKI, acute kidney injury; CKD, chronic kidney disease; ESRD, end stage renal disease.

^a^ The input key can be found in the eAppendix in Supplement 1.

The intervention costs were estimated based on the total time spent multiplied by hourly salary rate of all who participated in the monthly trainings. For interventions with surveillance, the cost of building and maintaining the digital dashboards during the trial period were also included. Intervention costs per patient were $12.74 (IQR, $9.56-$15.93) for collaborative with surveillance, $3.97 (IQR, $2.98-$4.96) for collaborative, $3.36 (IQR, $2.52-$4.20) for assistance with surveillance, and $2.69 (IQR, $2.02-$3.36) for assistance.

The utility associated with CKD and ESRD was estimated from the literature and expert opinion.^11^ The baseline utility for the mean patient included in this study, which is males aged 70 years with coronary heart disease without kidney dysfunction, was also estimated based on literature and expert opinion.^10^ The additional yearly costs associated with CKD and ESRD were based on 2020 reimbursement Medicare data.^13^ The cost estimates per episode of AKI were based on literature-derived values.^12^ Base case input values, 1-way sensitivity analysis variability range, and associated literature references can be found in Table 1.

### Statistical Analysis

#### Main Analysis

We used our Markov decision model to estimate costs adjusted to 2020 US dollars, using medical component of the consumer price indexes accounting for inflation, quality-adjusted life-years (QALYs) associated with each intervention and subsequent health states, as well as incremental cost-effectiveness ratio at a willingness-to-pay value of $100 000 per QALY.^14,15^ We chose a 3-year time horizon with annual cycles to reflect each strategies’ intermediate-term consequences.^7^ The perspective taken was that of Veteran Affairs, which provides care, operates facilities, and pays for all implementation costs. We discounted costs and QALYs at 3% per year.^16^ Data were analyzed from January to June 2024.

#### Sensitivity Analyses

We performed 1-way sensitivity analyses, individually varying parameters of different AKI rates of each strategy, transition probabilities, utilities, and costs. For AKI rates of each strategy and 30-day transition probabilities, we performed sensitivity analysis using 95% CIs from trial data as our range. We were not able to obtain 95% CI range for the yearly transition rates, which is based on the average of yearly transition rates for 3 years as well as the costs; thus, we varied the parameter plus and minus 25% from base case to demonstrate model sensitivity for these inputs. We also varied the cost inputs by plus and minus 25% for base case as well since we did not have reliable distribution data.

We performed a tornado diagram comparing the strategy with the highest intervention cost, collaborative with surveillance, with the strategy with the least expensive intervention cost, assistance, as the baseline for incremental net monetary benefit at a willingness-to-pay threshold of $100 000 per QALY. We also compared the collaborative with surveillance strategy with the assistance with surveillance strategy. Net monetary benefit (NMB) is a useful metric that uses willingness to pay threshold and converts health benefits into a common metric of dollars.^17^ It is calculated by the equation:

NMB = (effectiveness × willingness-to-pay) − cost

We also performed probabilistic sensitivity analysis using second-order Monte Carlo simulations to assess uncertainty in model parameters by drawing 1000 random samples for second-order uncertainty from each of the model parameter distribution.^18^ For most model inputs, we were able to use trial data or literature to obtain parameters, such as mean (SD), for distribution estimates. For inputs we did not have reliable data, we used conservative estimates for variance for determination of distribution. Furthermore, we used β distribution for all probabilities, β distribution for all utilities, and γ distribution for all costs.

#### Threshold Analysis

We performed threshold analysis on the intervention cost of the most cost-effective implementation strategy to ascertain how much the price of the intervention can increase before it is no longer the economically preferred strategy (eFigure 3 in Supplement 1). All analyses were performed using TreeAge Pro HealthCare 2024 (TreeAge).^19^

## Results

### Study Population

 Patient characteristics were balanced across 4 groups with an overall median (IQR) age of 70 (65-74) years, 119 119 males (97%), 25 789 Black patients (21%), 88 418 White patients (72%), and 8596 patients with other race or ethnicity (7%).^7^ Among 122 803 patients, 13 047 experienced AKIs (10.6%).

### Base Case Analysis

In the base case analysis, collaborative had a mean total cost per patient of $2480.32 and 2.31 total QALYs per person over the 3 years. Collaborative with surveillance strategy had a cost of $2958.87 and accumulated 2.30 QALYs, assistance had a cost of $3140.71 and 2.298 QALYs, and assistance with surveillance had a cost of $3223.08 and 2.298 QALYs gained. Collaborative with surveillance was cost-saving and economically preferred strategy among the 4 strategies (ie, was estimated to have the most QALYs gained, and the lowest total cost compared with all other interventions). All other strategies were dominated by collaborative with surveillance. Compared with assistance, collaborative with surveillance has a gain of 0.02 QALYs and $742.75 in cost savings (Table 2)

**Table 2.** Base Case Results of Cost-Effectiveness Analysis of Implementation Strategies for AKI Prevention Protocol

Strategy	Dominance	Cost, $	Incremental cost, $	QALYs	
				Effect outcome	Incremental effect outcome
Collaborative and surveillance	Dominant	2480.32	NA	2.314	NA
Assistance and surveillance	Dominated	2958.87	478.55	2.303	−0.011
Collaborative	Dominated	3140.71	660.39	2.299	−0.015
Assistance	Dominated	3223.08	742.75	2.297	−0.017

Abbreviation: QALY, quality-adjusted life years.

### Sensitivity Analysis

**Figure 2.**
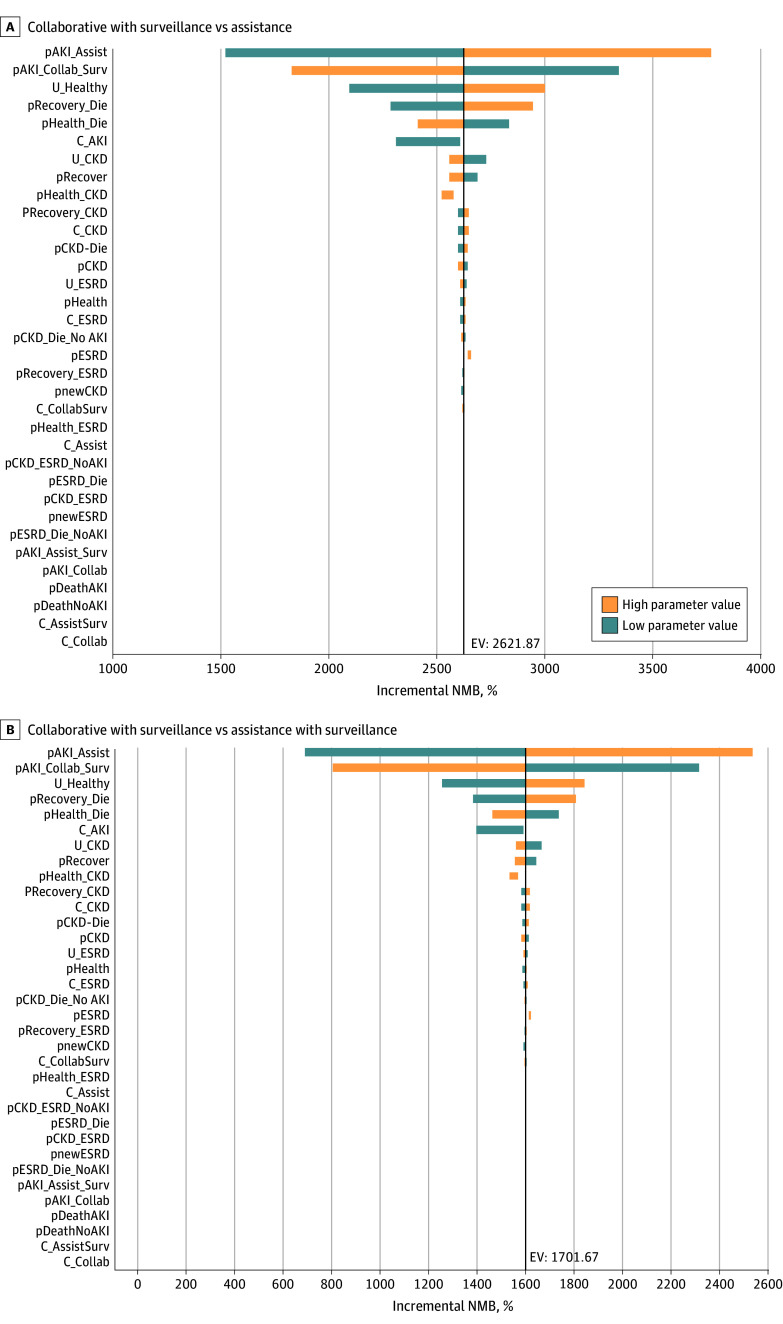
Tornado Diagram Comparing Collaborative With Surveillance vs Assistance C indicates cost in $; EV, expected value; NMB, Net Monetary Benefit; U, utilities in QALY. The input key can be found in eAppendix in Supplement 1.

**Figure 3.**
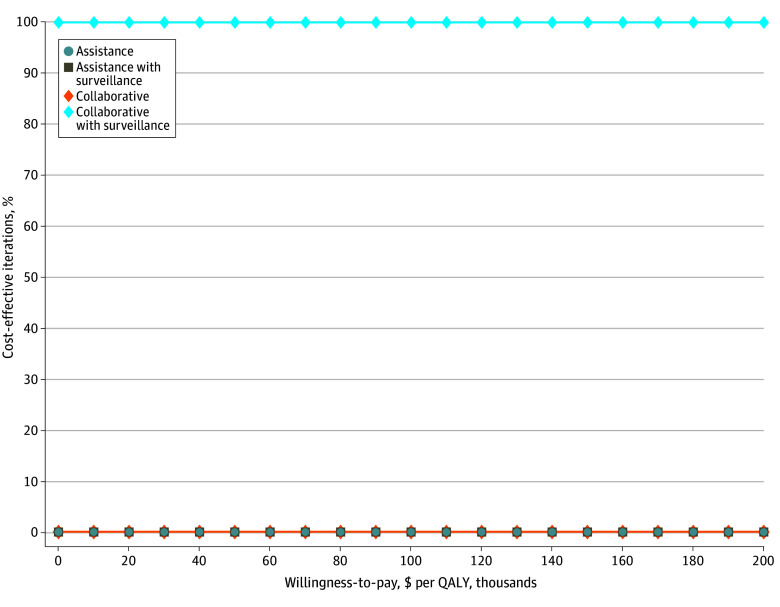
Cost Effectiveness Acceptability Curve Derived Using Probabilistic Sensitivity Analysis QALY indicates quality-adjusted life years.

In 1-way sensitivity analyses resulted in Collaborative with Surveillance being the preferred strategy. We performed a tornado diagram comparing collaborative with surveillance, the strategy with the highest upfront cost, with the least expensive, assistance, as the baseline for incremental net monetary benefit at a willingness-to-pay threshold of $100 000 per QALY (Figure 2). We also compared collaborative with surveillance with assistance with surveillance (Figure 2). Both tornado diagrams show that within the full range of variability for inputs, collaborative with surveillance remained the preferred strategy. The preferred strategy changes only when the AKI rate of other strategies falls below that of the collaborative with surveillance strategy. The cost associated with a strategy is primarily influenced by the costs associated with developing AKI, CKD, or ESRD rather than the upfront costs of the strategy. Therefore, the economically preferred strategy will be the environment that creates the lowest AKI rate, leading to lower subsequent rates of CKD, ESRD, and death. In probabilistic sensitivity analysis, with each model parameter varied simultaneously over prespecified distributions, collaborative with surveillance was preferred in 99.8% of model iterations (Figure 3).

### Threshold Analysis

We performed a threshold analysis on the intervention cost of the economically dominant strategy, collaboration with surveillance, to ascertain at what price point would it no longer be the preferred strategy. When collaboration with surveillance intervention costs rises above $1650.58, assistance with surveillance becomes the economically preferred strategy based on NMB with a WTP threshold of $100 000 per QALY (eFigure 3 in Supplement 1). At WTP threshold of $50 000 per QALY, the threshold for collaboration with surveillance intervention costs is $1080.17 per person.

When compared with assistance strategy, collaborative with surveillance remains the more cost-effective strategy until its intervention costs rises above $2544.12 per person at WTP threshold of $100 000 per QALY and at a price of $1664.06 per person at WTP threshold of $50 000 per QALY.

## Discussion

In this Markov model-based cost-effectiveness analysis, collaborative, team-base virtual intervention with periodic communication of outcomes, and ongoing surveillance was the most effective and most cost saving strategy for AKI prevention protocol implementation for VAMC patients undergoing cardiac catheterization. The wide variation of 1-way and probabilistic sensitivity analyses across the most clinically relevant scenarios did not alter the results of the optimal strategy.

The IMPROVE AKI trial successfully demonstrated that using team-based training approaches that leverage quality improvement methods and performance feedback via digital reports was effective in the implementation of a new protocol for the prevention of contrast-associated AKIs. However, this strategy required significant investment, which in this study consisted of 18-monthly multidisciplinary team virtual meetings with dedicated training on quality improvement methodologies, such as Six Sigma, Lean,and PDSA cycles. This contrasts with the more traditional implementation strategy of technical assistance, which consists of teaching and assisting with the implementation of the desired protocol alone with no quality improvement training and minimal subsequent team-based collaboration. Our study found that from the perspective of the Veterans Affairs health care system, this added effort, cost, and time used to teach and use quality improvement methods in team-based collaboratives reinforced with regular feedback to the teams by dedicated surveillance reporting generated the greatest reductions in AKI and subsequent cost-savings for the medical centers.

Our analysis demonstrates the greater impact of reducing AKI compared with the per-patient costs of implementing almost any strategy that improves guideline-based care. AKI and the subsequent development of CKD and ESRD overwhelm the wide range of realistic implementation costs associated with health care team-based interventions. The observed costs were attributed primarily to the cost of training, which comes out to be relatively small amount per hospital (eFigure 2 in Supplement 1) and even less costly per patient as noted in Table 1. The most expensive study site from the trial for collaborative with surveillance cost $6402 for the entire intervention. The intervention was cheaper than 1 episode of AKI, which was estimated to cost an additional $14 000 on average per patient. Our threshold analysis reveals that when compared with assistance, collaborative with surveillance can cost up to $2544.12 per person at a willingness-to-pay threshold of $100 000 per QALY gained to remain the more economically preferred strategy. When compared with assistance with surveillance, the price threshold for collaborative with surveillance to remain the preferred strategy is $1650.58 per person, which is significantly more than the actual intervention cost of $12.74 (IQR, $9.56-$15.93) per person.

This study demonstrated a significant incentive to provide the collaborative with surveillance implementation strategy to all health care organizations or payors that would be responsible for both the cardiac catheterization procedure and subsequent patient care. Furthermore, these promising findings may be generalizable to other endovascular procedures, such as cardiac valve repair, revascularization of peripheral vascular disease, aneurysmal repair, and others with a high incidence of contrast induced AKIs. In addition, collaborative with surveillance was not designed uniquely for AKI prevention protocol, and thus, this implementation strategy could be evaluated and used for the implementation of other complex practice change protocols. Systematic, virtual learning approaches for health care implementation are particularly exciting considering the evidence for protocol-driven safety and quality initiatives with regular feedback; most notable examples being the implementation of safe surgery checklists and time outs.^20^ The strength of the study relies on the use of detailed trial data. This helped in the accuracy of assumptions and parameter estimates.

### Limitations

This study has limitations. Setting our study at VAMCs cwould impact the generalizability of this study. VAMC is a single-payer, government-run health care system. We performed this evaluation in the perspective of the medical centers, and costs and cost-savings were assumed to be taken on by the medical centers. However, this is not the case outside of the single-payer VAMC setting, and reimbursements through third-party payers for private hospitals would not result in the same cost implications seen with VAMCs. The costs associated with AKI and kidney disease in private hospital settings would most appropriately be viewed from societal and patient perspectives. Of note, the VAMC patient population is homogenous, as was the case in this study, where over 90% of patients were male, with a majority being White individuals, which would not be representative of other hospital systems. In addition, VA patients have been shown to have unfavorable differences in sociodemographic status, health status, and medical resource use compared with general patient population.^21^

Furthermore, since we limited our study to a 3-year time horizon to reflect trial observations, our analysis also limits the long-term evaluation of strategies. While on 1 hand, a longer horizon should theoretically amplify the cost and effectiveness benefit of our intervention decreasing incidence of kidney disease; on the other hand, the impact of the intervention may fade over time, particularly without repeat training. Our modeling of the immediate impact of AKIs was also limited. We added an additional $14 000 per episode of AKI per person based on literature estimates, but the actual immediate impact varies greatly based on patient specific factors. As AKI costs drove our results, this cost needs the most accurate estimation in future trials or when being implemented at individual institutions. In addition, while we our model had a great deal of fidelity due to having the data from a large trial with known 95% CIs for many of the probabilities to use in our sensitivity analyses, we did have to use ±25% for some of the inputs for our 1-way sensitivity analyses and best estimates of SDs for our probabilistic sensitivity analyses.

Additionally, while ensuring adequate intravenous volume and reduction in contrast volume were the focus of the protocols implemented in the IMPROVE AKI study, there are many additional and varied evidence-based strategies available in the prevention of contrast-induced AKIs among those undergoing cardiac catheterization. For an example, while IMPROVE AKI used a standardized approach for hydration, there have been evidence supporting individualized hydration that is tailored to patients’ individual risk and volume status.^22^ Other major prevention strategies for prevention of contrast induced AKI in cardiac catheterization is the preferential use of radial access over femora access as well as routine use of AKI risk calculators for all patients.^23^ An important point to note is that the IMPROVE AKI study and this cost-effectiveness study primarily evaluated the different strategies for implementation of AKI prevention protocols, while prior foundation studies evaluated the association of the prevention protocols themselves. Interestingly, the most effective and cost-saving implementation strategy, collaborative with surveillance, includes multidisciplinary team meetings to discuss new AKI prevention evidence and implementation strategies to continue improving their monitored outcomes. Therefore, ideally, this implementation strategy would lead to modification of AKI prevention protocols to include the most up-to-date best practices.

## Conclusions

The findings of this economic analysis of implementation of AKI prevention strategies suggest that using quality improvement methods in team-based settings with performance feedback for the implementation of AKI prevention protocols was both effective and cost-saving. This promising implementation strategy should be evaluated further in other endovascular procedures and other protocol and checklist implementation efforts.

## Notes

Supplement 1. **eFigure 1.** Decision Tree **eFigure 2.** Costs Shown Represents the Most Expensive Sites for Each Intervention **eFigure 3.** Threshold Analysis on Intervention Cost of Collaboration With Surveillance **eTable.** CHEERS Checklist **eAppendix.** Input Key

Supplement 2.
Data Sharing Statement

## References

[1] Benjamin EJ, Blaha MJ, Chiuve SE, ; American Heart Association Statistics Committee and Stroke Statistics Subcommittee. Heart disease and stroke statistics-2017 update: a report from the American Heart Association. Circulation. 2017;135(10):e146-e603. doi:10.1161/CIR.0000000000000485 28122885 PMC5408160

[2] Chertow GM, Burdick E, Honour M, Bonventre JV, Bates DW. Acute kidney injury, mortality, length of stay, and costs in hospitalized patients. J Am Soc Nephrol. 2005;16(11):3365-3370. doi:10.1681/ASN.2004090740 16177006

[3] Brown JR, Malenka DJ, DeVries JT, . Transient and persistent renal dysfunction are predictors of survival after percutaneous coronary intervention: insights from the Dartmouth Dynamic Registry. Catheter Cardiovasc Interv. 2008;72(3):347-354. doi:10.1002/ccd.21619 18729173 PMC6643288

[4] James MT, Samuel SM, Manning MA, . Contrast-induced acute kidney injury and risk of adverse clinical outcomes after coronary angiography: a systematic review and meta-analysis. Circ Cardiovasc Interv. 2013;6(1):37-43. doi:10.1161/CIRCINTERVENTIONS.112.974493 23322741

[5] Ali A, Bhan C, Malik MB, Ahmad MQ, Sami SA. The Prevention and management of contrast-induced acute kidney injury: a mini-review of the literature. Cureus. 2018;10(9):e3284. doi:10.7759/cureus.3284 30443454 PMC6235634

[6] Brown JR, McCullough PA, Splaine ME, ; Northern New England Cardiovascular Disease Study Group. How do centres begin the process to prevent contrast-induced acute kidney injury: a report from a new regional collaborative. BMJ Qual Saf. 2012;21(1):54-62. doi:10.1136/bmjqs-2011-000041 21890755 PMC4872501

[7] Brown JR, Solomon R, Stabler ME, . Team-based coaching intervention to improve contrast-associated acute kidney injury: a cluster-randomized trial. Clin J Am Soc Nephrol. 2023;18(3):315-326. doi:10.2215/CJN.0000000000000067 36787125 PMC10103221

[8] Barr E, Brannan GD. Quality Improvement Methods (LEAN, PDSA, SIX SIGMA). *StatPearls*. Published online January 11, 2024. Accessed May 2, 2024. https://www.ncbi.nlm.nih.gov/books/NBK599556/38261708

[9] Brown JR, Solomon RJ, Sarnak MJ, ; Northern New England Cardiovascular Disease Study Group. Reducing contrast-induced acute kidney injury using a regional multicenter quality improvement intervention. Circ Cardiovasc Qual Outcomes. 2014;7(5):693-700. doi:10.1161/CIRCOUTCOMES.114.000903 25074372 PMC4869689

[10] Matza LS, Stewart KD, Gandra SR, . Acute and chronic impact of cardiovascular events on health state utilities. BMC Health Serv Res. 2015;15(1):1-11. doi:10.1186/s12913-015-0772-9 25896804 PMC4408571

[11] Cooper JT, Lloyd A, Sanchez JJG, Sörstadius E, Briggs A, McFarlane P. Health related quality of life utility weights for economic evaluation through different stages of chronic kidney disease: a systematic literature review. Health Qual Life Outcomes. 2020;18(1):310. doi:10.1186/s12955-020-01559-x 32957990 PMC7507735

[12] Subramanian S, Tumlin J, Bapat B, Zyczynski T. Economic burden of contrast-induced nephropathy: implications for prevention strategies. J Med Econ. 2007;10(2):119-134. doi:10.3111/200710119134 19702434

[13] Annual Data Report. US Renal Data System. Accessed May 1, 2024. https://usrds-adr.niddk.nih.gov/2022/chronic-kidney-disease/6-healthcare-expenditures-for-persons-with-ckd

[14] Databases, tables, and calculators by subject. US Bureau of Labor Statistics. Accessed May 7, 2024. https://data.bls.gov/timeseries/CUSR0000SAM?output_view=pct_1mth

[15] Vanness DJ, Lomas J, Ahn H. A Health opportunity cost threshold for cost-effectiveness analysis in the United States. Ann Intern Med. 2021;174(1):25-32. doi:10.7326/M20-1392 33136426

[16] Sanders GD, Neumann PJ, Basu A, . Recommendations for conduct, methodological practices, and reporting of cost-effectiveness analyses: second panel on cost-effectiveness in health and medicine. JAMA. 2016;316(10):1093-1103. doi:10.1001/jama.2016.12195 27623463

[17] Klarenbach S, Cameron C, Singh S, Ur E. Cost-effectiveness of second-line antihyperglycemic therapy in patients with type 2 diabetes mellitus inadequately controlled on metformin. CMAJ. 2011;183(16):E1213-E1220. doi:10.1503/cmaj.110178 21969406 PMC3216433

[18] Briggs AH, Weinstein MC, Fenwick EAL, Karnon J, Sculpher MJ, Paltiel AD; ISPOR-SMDM Modeling Good Research Practices Task Force. Model parameter estimation and uncertainty analysis: a report of the ISPOR-SMDM Modeling Good Research Practices Task Force Working Group-6. Med Decis Making. 2012;32(5):722-732. doi:10.1177/0272989X12458348 22990087

[19] TreeAge Pro. TreeAge Software. Accessed February 21, 2025. http://www.treeage.com

[20] Papadakis M, Meiwandi A, Grzybowski A. The WHO safer surgery checklist time out procedure revisited: strategies to optimise compliance and safety. Int J Surg. 2019;69:19-22. doi:10.1016/j.ijsu.2019.07.006 31310820

[21] Agha Z, Lofgren RP, VanRuiswyk JV, Layde PM. Are patients at Veterans Affairs medical centers sicker: a comparative analysis of health status and medical resource use. Arch Intern Med. 2000;160(21):3252-3257. doi:10.1001/archinte.160.21.3252 11088086

[22] Moroni F, Baldetti L, Kabali C, . Tailored versus standard hydration to prevent acute kidney injury after percutaneous coronary intervention: network meta-analysis. J Am Heart Assoc. 2021;10(13):e021342. doi:10.1161/JAHA.121.021342 34169747 PMC8403299

[23] Quality initiatives for prevention of contrast-induced acute kidney injury. Society for Cardiovascular Angiography and Interventions. Accessed December 14, 2024. https://scai.org/quality-initiatives-prevention-contrast-induced-acute-kidney-injury

