# Supplementary material for: Cost-Effectiveness of Team-Based Coaching With Surveillance for Prevention of Acute Kidney Injuries
Source: JAMA Netw Open. 2025 Apr 2;8(4):e252503. doi: 10.1001/jamanetworkopen.2025.2503 (PMC11966327; doi:10.1001/jamanetworkopen.2025.2503)
Supplement: Supplement 1. — eFigure 1. Decision Tree eFigure 2. Costs Shown Represents the Most Expensive Sites for Each Intervention eFigure 3. Threshold Analysis on Intervention Cost of Collaboration With Surveillance eTable. CHEERS Checklist eAppendix. Input Key [file jamanetwopen-e252503-s001.pdf]

Supplemental Online Content

Xiao D, Davis SE, Godfrey CM, et al. Cost-effectiveness of team-based coaching with surveillance for prevention of acute kidney injuries. *JAMA Netw Open*. 2025;8(4):e252503. doi:10.1001/jamanetworkopen.2025.2503

eFigure 1. Decision Tree
eFigure 2. Costs Shown Represents the Most Expensive Sites for Each Intervention
eFigure 3. Threshold Analysis on Intervention Cost of Collaboration With Surveillance
eTable. CHEERS Checklist
eAppendix. Input Key

This supplemental material has been provided by the authors to give readers additional information about their work.

© 2025 Xiao D et al. *JAMA Network Open*.

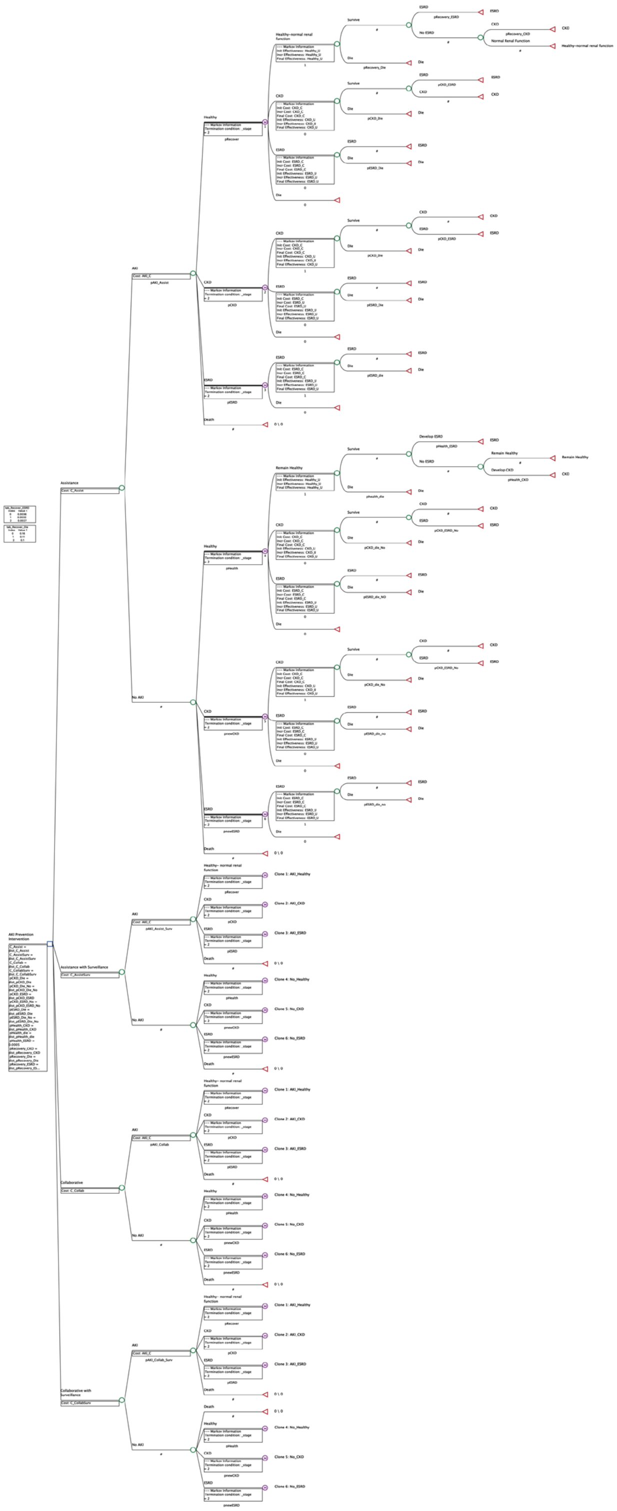

© 2025 Xiao D et al. *JAMA Network Open*.

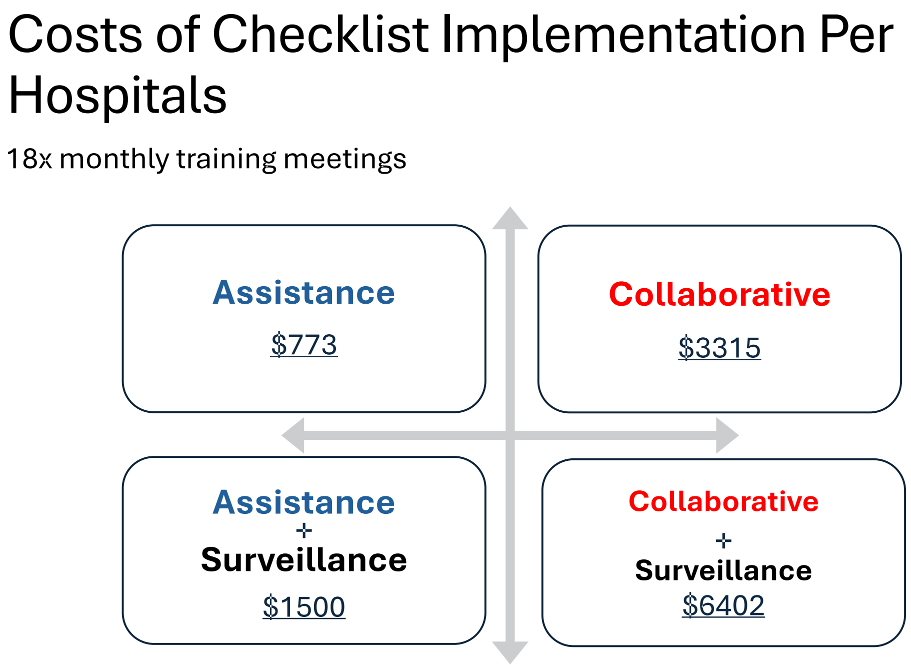

Supplement Figure S1: Costs shown represents the most expensive sites for each intervention.

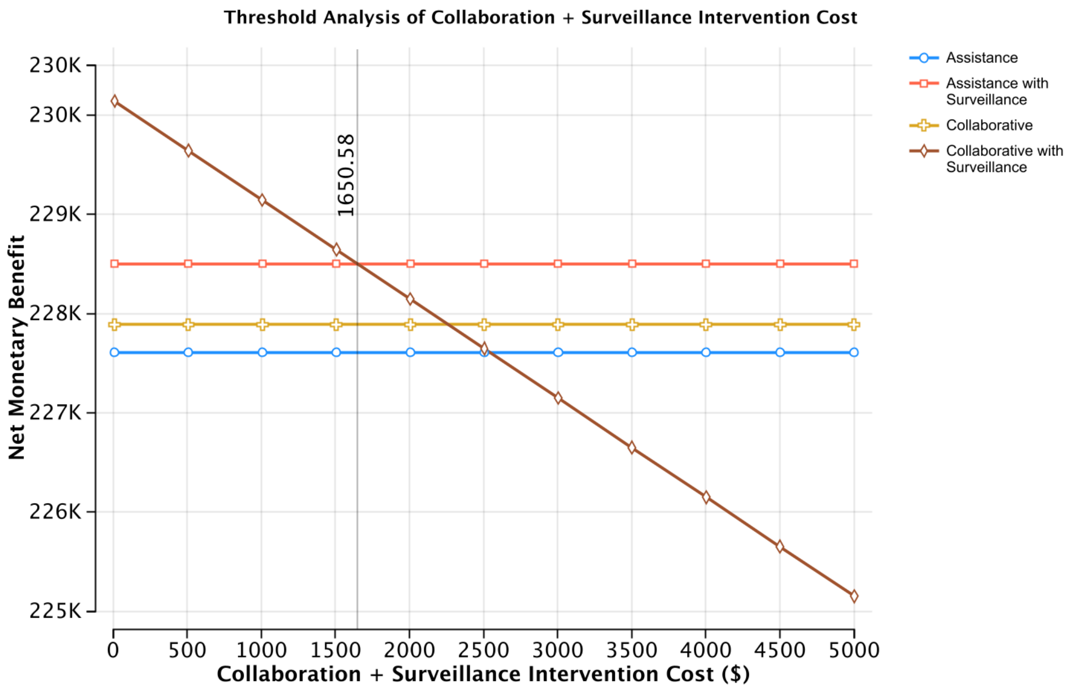

Supplement Figure S2: Threshold Analysis on Intervention Cost of Collaboration with Surveillance

© 2025 Xiao D et al. *JAMA Network Open*.

CHEERS Checklist

	Item	Guidance for Reporting	Reported insection
**TITLE**			
Title	1	Identify the study as an economic evaluation and specify theinterventions being compared.	Page 1
**ABSTRACT**			
Abstract	2	Provide a structured summary that highlights context, key methods,results and alternative analyses.	Page 2
**INTRODUCTION**			
Background and objectives	3	Give the context for the study, the study question and its practicalrelevance for decision making in policy or practice.	Page 3-4
**METHODS**			
Health economic analysis plan	4	Indicate whether a health economic analysis plan was developed and where available.	Page 3-4
Study population	5	Describe characteristics of the study population (such as age range, demographics, socioeconomic, or clinical characteristics).	Page 3-4
Setting and location	6	Provide relevant contextual information that may influence findings.	Page 3-4
Comparators	7	Describe the interventions or strategies being compared and why chosen.	Page 3-4
Perspective	8	State the perspective(s) adopted by the study and why chosen.	Page 3-4
Time horizon	9	State the time horizon for the study and why appropriate.	Page 3-4
Discount rate	10	Report the discount rate(s) and reason chosen.	Page 3-4
Selection of outcomes	11	Describe what outcomes were used as the measure(s) of benefit(s)and harm(s).	Page 3-4
Measurement of outcomes	12	Describe how outcomes used to capture benefit(s) and harm(s)were measured.	Page 3-4
Valuation of outcomes	13	Describe the population and methods used to measure and value outcomes.	Page 3-4
Measurement and valuation of resources and costs	14	Describe how costs were valued.	Page 3-4
Currency, price date, and conversion	15	Report the dates of the estimated resource quantities and unit costs, plus thecurrency and year of conversion.	Page 3-4
Rationale and description of model	16	If modelling is used, describe in detail and why used. Report if the model is publicly available and where it can be accessed.	Page 3-4
Analytics and assumptions	17	Describe any methods for analysing or statistically transforming data, anyextrapolation methods, and approaches for validating any model used.	Page 3-4
Characterizing heterogeneity	18	Describe any methods used for estimating how the results of the studyvary for sub-groups.	Page 3-4
Characterizing distributional effects	19	Describe how impacts are distributed across different individuals or adjustments made to reflect priority populations.	NA
Characterizing uncertainty	20	Describe methods to characterize any sources of uncertainty in the analysis.	Page 3-4
Approach to engagement with patients and others affected by the study	21	Describe any approaches to engage patients or service recipients, the general public, communities, or stakeholders (e.g., clinicians or payers) inthe design of the study.	NA
**RESULTS**			
Study parameters	22	Report all analytic inputs (e.g., values, ranges, references) includinguncertainty or distributional assumptions.	Page 3-4 (table1)
Summary of main results	23	Report the mean values for the main categories of costs and outcomes ofinterest and summarise them in the most appropriate overall measure.	Page 6
Effect of uncertainty	24	Describe how uncertainty about analytic judgments, inputs, or projections affect findings. Report the effect of choice of discount rate and time horizon,if applicable.	Page 6
Effect of engagement with patients and othersaffected by the	25	Report on any difference patient/service recipient, general public, community,or stakeholder involvement made to the approach or	NA

© 2025 Xiao D et al. *JAMA Network Open*.

study		findings of the study	
**DISCUSSION**			
Study findings, limitations, generalizability, and current knowledge	26	Report key findings, limitations, ethical or equity considerations not captured,and how these could impact patients, policy, or practice.	Page 7-8
**OTHER RELEVANT INFORMATION**			
Source of funding	27	Describe how the study was funded and any role of the funder inthe identification, design, conduct, and reporting of the analysis	Page 1
Conflicts of interest	28	Report authors conflicts of interest according to journal or International Committee of Medical Journal Editors requirements.	Page 1

Supplement Table S1: CHEERS Checklist

© 2025 Xiao D et al. *JAMA Network Open*.

eAppendix. Input Key

pAKI_Assist: probability of acute kidney injuries in Technical Assistance strategy
pAKI_Collab_Surv: probability of acute kidney injuries in Collaborative with Surveillance strategy
U_Healthy: utility of average patient in study without renal injury
pRecovery_Die: probability of death after renal recovery from acute renal injury
pHealth_Die: probability of death for average patient in study without renal injury
C_AKI: cost in dollars per episode of acute kidney injury
U_CKD: utility of average patient in study with chronic kidney disease
pRecover: probability of renal recovery from acute kidney injury
pHealth_CKD: probability of developing chronic kidney disease without acute kidney injury
PRecovery_CKD: probability of developing chronic kidney disease after renal recovery from acute kidney injury
C_CKD: cost in dollars per year associated with chronic kidney disease
pCKD_Die: probability of death in patients with chronic kidney disease
pCKD: probability of developing CKD after acute kidney injury
U_ESRD: utility of average patient in study with end stage renal disease
pHealth: probability of remaining renally healthy without acute kidney injury
C_ESRD: cost in dollars per year associated with end stage renal disease
pCKD_Die_No AKI: probability of death with chronic kidney disease and no acute kidney injury
pESRD: probability of developing end stage renal disease after acute kidney injury
pRecovery_ESRD: probability of developing end stage renal disease after renal recovery from acute kidney injury
pnewCKD: probability of developing chronic kidney disease without acute kidney injury
C_CollabSurv: cost in dollars associated with Collaborative with Surveillance strategy
pHealth_ESRD: probability of developing end stage renal disease without acute kidney injury
C_Assist: cost in dollars associated with Technical Assistance strategy
pCKD_ESRD_NoAKI: probability of developing end stage renal disease in patients with chronic kidney disease and no acute kidney injury
pESRD_Die: probability of death in patients with end stage renal disease
pCKD_ESRD: probability of developing end stage renal disease in patients with chronic kidney disease after acute kidney injury
pnewESRD: probability of developing end stage renal disease in renally healthy patients without acute kidney injury
pESRD_Die_NoAKI: probability of death in patients with end stage renal disease without acute kidney injury
pAKI_Assist_Surv: probability of acute kidney injuries in Technical Assistance with Surveillance strategy
pAKI_Collab: probability of acute kidney injuries in Collaborative strategy
pDeathAKI: probability of death after acute kidney injury
pDeathNoAKI: probability of death without acute kidney injury
C_AssistSurv: cost in dollars associated with Technical Assistance with Surveillance strategy
C_Collab: cost in dollars associated with Collaborative strategy

© 2025 Xiao D et al. *JAMA Network Open*.
