# Supplementary material for: Cost-Effectiveness of Team-Based Coaching With Surveillance for Prevention of Acute Kidney Injuries
Source: JAMA Netw Open. 2025 Apr 2;8(4):e252503. doi: 10.1001/jamanetworkopen.2025.2503 (PMC11966327; doi:10.1001/jamanetworkopen.2025.2503)
Supplement: Supplement 2. — Data Sharing Statement [file jamanetwopen-e252503-s002.pdf]

Data Sharing Statement

Xiao. Cost-Effectiveness of Team-Based Coaching With Surveillance for Prevention of Acute Kidney Injuries. *JAMA Netw Open*. Published April 02, 2025.
doi:10.1001/jamanetworkopen.2025.2503

Data
Data available: Yes
Data types: Deidentified participant data
How to access data: Data can be obtained upon request by contacting corresponding author.
When available: With publication

Supporting Documents
Document types: Statistical/analytic code
How to access documents: Data can be obtained upon request by contacting corresponding author.
When available: With publication

Additional Information
Who can access the data: By anyone requesting the data
Types of analyses: For any purpose
Mechanisms of data availability: With investigator support.
